# Hotspot model shows how location-based superspreading accelerates and reshapes epidemics

**DOI:** 10.1093/pnasnexus/pgaf299

**Published:** 2025-09-22

**Authors:** Brendan Wallace, Dobromir Dimitrov, Laurent Hébert-Dufresne, Andrew M Berdahl

**Affiliations:** Quantitative Ecology and Resource Management, University of Washington, Seattle, WA 98195, USA; School of Aquatic and Fishery Sciences, University of Washington, Seattle, WA 98195, USA; Department of Applied Mathematics, University of Washington, Seattle, WA 98195, USA; Department of Applied Mathematics, University of Washington, Seattle, WA 98195, USA; Vaccine and Infectious Disease Division, Fred Hutchinson Cancer Center, Seattle, WA 98109, USA; Vermont Complex Systems Institute, University of Vermont, Burlington, VT 05405, USA; Department of Computer Science, University of Vermont, Burlington, VT 05405, USA; Santa Fe institute, Santa Fe, NM 87501, USA; Quantitative Ecology and Resource Management, University of Washington, Seattle, WA 98195, USA; School of Aquatic and Fishery Sciences, University of Washington, Seattle, WA 98195, USA

**Keywords:** epidemiology, heterogeneity, superspreading events, risk structure, fission–fusion

## Abstract

During outbreaks of many diseases, a small number of infected individuals are responsible for a disproportionately large number of new infections in what are called superspreading events (SSEs). SSEs broadly fall into four categories: (i) a single individual is more infectious due to biological differences in their infection or (ii) their greater degree of social connection; or (iii) the disease spreads more readily in certain high-risk facilities or (iv) “opportunistic” situations such as large gatherings. Existing modeling approaches work well to understand the first two of these but are not well suited to describe the dynamics in the latter two. Here, we introduce a simple agent-based model which captures the essential features of disease spreading more readily at high-risk locations or gatherings, which we call “hotspots.” In our model, disease spreads and people recover as in a standard Susceptible, Infected, Recovered model, but agents are also characterized by individual probability of visiting the hotspot where disease spreads much more readily, providing an additional risk structure to the population. We use this model to investigate how an outbreak’s probability, peak, and final size all vary under different risk heterogeneity assumptions. We show how some particular distributions of risk-taking behavior across the population heighten these effects. We complement our simulations with analytic results that provide theoretical bases for all of our numerical results and allow for robust interpretation and prediction.

Significance Statement Models of disease spread often treat people and locations homogeneously. In reality, disease spreads more readily in some “hotspot” locations and some people visit these locations more often, potentially leading to superspreading events. We modified a classic model of disease spread to incorporate heterogeneous disease spread in different locations and diversity in risk-taking behavior in different people. Simulations and mathematical results from our simple model agree with established understanding of “superspreading” in its assessment of the probability of large outbreaks taking place but predict some notable departures related to the size and overall development of an epidemic.

## Introduction

After the SARS pandemic in 2005, researchers noted that disease transmission was not homogeneous: some small number of infected individuals became foci of superspreading events responsible for a disproportionately large number of total infections (1). Subsequent research noted a similar pattern in other diseases: 15 to 20% of infected individuals caused 75 to 85% of subsequent infections in diseases as diverse as measles, smallpox, monkeypox, HIV, and others (2, 3). The COVID-19 pandemic followed a similar pattern: transmission is dominated by superspreading events (SSEs) in which a superspreading individual infects many people over a short time (4, 5). By identifying and understanding the dynamics of superspreading, public health interventions can be targeted more effectively.

A recent review of superspreading in the context of SARS-CoV-2 identified at least four main types of SSEs (6). (i) Individuals might be more infectious for biological or physiological reasons. Viral loads depend on time since infection, where the infection was initiated (e.g. lower or upper respiratory tract (7)), immune history, and potentially even demographics (8). (ii) Certain individuals simply have more contacts than others and can therefore infect more people. Contact patterns are known to vary greatly by age and profession (9). (iii) High-risk SSEs are found in confined locations where individuals are *consistently* placed in settings with high transmission risks. These SSEs have been covered extensively in media and studies alike, with classic examples found in meat processing plants (10), prisons (11), or long-term care facilities (12) for example. (iv) Opportunistic SSEs occur when a large number of people *temporarily* gather together and increase local transmission risks through an activity like singing or shouting (13). Many such SSEs have been reported at nightclubs, restaurants, cruise ships, choirs, and other large events (14).

These four types of SSEs can be divided in two general classes, which have received disparate attention in modeling efforts. Indeed, the first two types of super-spreading events stem from individual factors which are either biological or social, respectively. These effects can be naturally included in standard compartmental or network epidemic models. For instance, age-based differences in contact rates and susceptibility were quickly incorporated in mathematical models of COVID-19 to inform interventions aimed at constraining or relaxing contact patterns (15–17). Compartmental models can be used to account for heterogeneous populations where a discrete number of classes of individuals are used to account for risk factors (18, 19). Perhaps more generally, weighted contact networks can help capture individual differences in social behavior and contact patterns (20–22). Finally, data-driven simulations based on individual mobility data can also capture how specific individuals and locations lead to a majority of infections (23, 24).

The second class of SSEs deals not with individual factors but with groups or context determined by location, activity, duration, and repetition of gatherings (25). High-risk SSEs are found in settings where many of the same individuals are repeatedly exposed to potential transmissions; whereas opportunistic SSEs are very similar but more ephemeral and less correlated over time. They can be summarized as social fission–fusion dynamics of groups forming and disbanding (26–28) and are mostly distinguished by their correlation patterns from one group to the next. The important effects of this fission–fusion dynamics can be included in large network or agent-based models based on temporal mobility data at geographical points of interest (23). However, given the focus of network models on individual behaviors, we have fewer analytical tools and abstract models built specifically for the study of group-based dynamics.

We propose to study this class of context-based SSEs under a unified framing of “hotspot” transmission that includes temporal and individual correlations through agent-based risk distributions. Consider a town with a large meat-packing plant as its largest employer, or a city with a vibrant nightclub culture. In these populations, the distribution of how frequently individuals visit these hotspots will drive their epidemic risk or exposure to possible transmissions. The resulting fission–fusion dynamics will be the main driver of the local epidemic dynamics.

Fission–fusion dynamics created by people using hotspots bridges the gap between classic well-mixed or mass-action epidemic models and more realistic data-driven simulations. Our aim is to employ enough complexity to capture risk heterogeneity while remaining general enough to be analytically tractable. To this end, we introduce an agent-based model with a simple risk structure and location-based effects. Agents in the model are characterized by their individual frequency of visiting a hotspot location where disease spreads more readily. We use this model to investigate how an outbreak’s probability, peak, and final size all vary under different heterogeneity assumptions. We show how some particular distributions of risk-taking behavior across the population heighten these effects. We complement our simulations with analytic results that provide theoretical bases for all of our numerical results and allow for robust interpretation and prediction.

### Agent-based hotspot SIR model

**Fig. 1.**
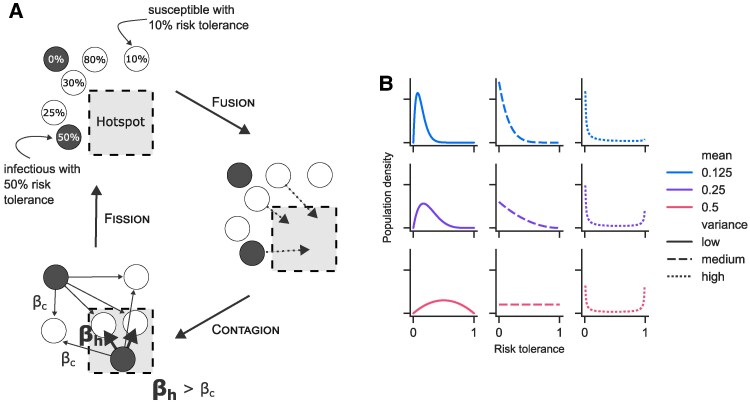
Hotspot model. A) The model assigns each individual a fixed risk tolerance value between 0 and 1. Each day, individuals visit the hotspot with probability equal to their risk tolerance. Infected agents in the hotspot spread the disease with high transmission rate to susceptible agents in the hotspot. At the same time, all infected agents spread the disease to all susceptible agents with low transmission rate. Infected agents eventually recover and cannot be infected again, and the simulation ends when no agents are infected. B) We consider different risk tolerance distributions for the population using beta distributions with low, medium, and high mean; and low, medium, and high variance. See Supplementary material for more details.

Our hotspot SIR (hsSIR) model simulates *N* individuals in a fixed population, in which individuals can be susceptible (S), infected (I), or recovered (R) (Fig. 1). Each time step (day), each infected agent *i* may transmit an infection to any susceptible agent with fixed probability $\beta_{c}$ (homogeneous community spread). Additionally, each agent independently visits a location with increased transmission (the “hotspot”) with probability $\rho_{i}$ (risk tolerance); where disease spreads from each infected individual to each susceptible individual with elevated probability $\beta_{h}$ (hotspot spread). We explore scenarios with different proportion of transmission (25–75%) occurring in the hotspot in comparison with the homogeneous scenario (0%). Individuals recover from infection after a fixed number of days *D*. We start each simulation by choosing one individual at random to be infected and continue until all agents are either susceptible or recovered. The probability to visit the hotspot $\rho_{i}$ is fixed for each individual but varies between individuals.

In our numerical experiments, we consider diseases with different distributions of risk tolerance $\rho_{i}$ across the population (see Fig. 1B), and different levels of community ($\beta_{c}$) and hotspot ($\beta_{h}$) spread. We calculate basic and effective transmission rates $R_{0}$ and $R_{t}$, which are the expected number of secondary infections per infection at the beginning of an outbreak and any time during an outbreak, respectively. To isolate the impacts of hotspot dynamics, we fix $R_{0}$ across multiple scenarios while allowing individual transmission rates (which in practice may be more difficult to measure than $R_{0}$ or $R_{t}$) to vary. See Table 1 for a list and description of model parameters, dynamic variables and calculated quantities, and the Tables S1 and S2 for a full description of how parameters are set.

**Table 1.** Parameter values, variables, and quantities used in simulations.

Type	Symbol	Description	{Value/Equ.}
Parameters	$\beta_{h}$	Hotspot spread rate	[0,0.384]
	$\beta_{c}$	Community spread rate	[0,0.008]
	*D*	Recovery time	[1,8]
	*N*	Total number of individuals	1,000
	$\rho_{i}$	Risk tolerance of individual i	[0,1]
Dynamic	**S**	Set of susceptible individuals	
variables	**I**	Set of infected individuals	
	**R**	Set of recovered individuals	
Calculated	S, I, R	Number of individuals in **S**, **I**, **R**	|S| , |I|, |R|
quantities	$\bar{\rho }$	Mean risk-tolerance	$\sum_{i}\rho_{i}/N$
	${\bar{\rho }}_{S}$	…(susceptible only)	$\sum_{i\in S}\rho_{i}/S$
	${\bar{\rho }}_{I}$	…(infected only)	$\sum_{i\in I}\rho_{i}/I$
	Var(ρ)	Variance of risk tolerance	$\sum_{i}(\rho_{i}-\bar{\rho }{)}^{2}/N$
	$\text{Var}(\rho_{S})$	…(susceptible only)	$\sum_{i\in S}(\rho_{i}-{\bar{\rho }}_{S}{)}^{2}/S$
	$\text{Var}(\rho_{I})$	…(infected only)	$\sum_{i\in I}(\rho_{i}-{\bar{\rho }}_{I}{)}^{2}/I$
	$R_{0}$	Basic reproduction number	$D({\bar{\rho }}^{2}\beta_{h}+\beta_{c})N$
	$R_{e}$	Effective reproduction number	$D({\bar{\rho }}_{I}{\bar{\rho }}_{S}\beta_{h}+\beta_{c})S$
	$f_{h}$	Hotspot spread fraction	$D{\bar{\rho }}^{2}\beta_{h}N/R_{0}$

## Results

### Large outbreaks are less likely with hotspot spread

**Fig. 2.**
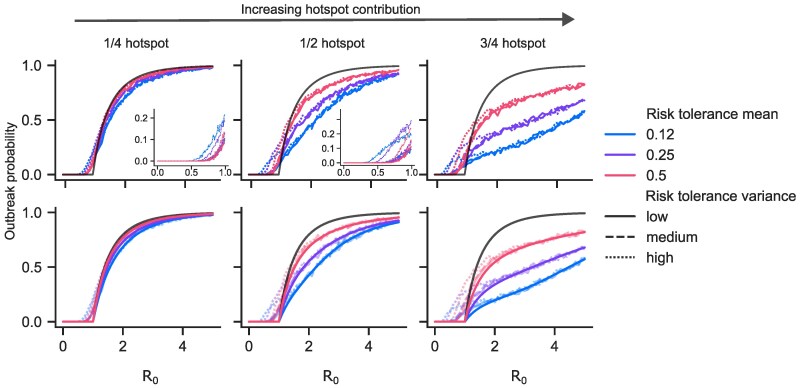
Outbreak probability. We say that an outbreak has occurred if at least 5% of individuals were infected over the course of a simulation. In a homogeneous model, the probability of this occurring increases sharply as a function of $R_{0}$ (black lines). (Top) Colored lines show outbreak probability across 1,000 trials in the agent-based model as a function of $R_{0}$ for nine different risk tolerance distributions; different columns show varying contributions of hotspot spread to $R_{0}$; insets show outbreak probability for low $R_{0}$. (Bottom) Agent-based probabilities are now shown faded, with theoretical outbreak probabilities overlaid in solid lines, showing excellent agreement for $R_{0}$ above 1 but an underestimate of outbreak probability for $R_{0}$ below 1.

Our hotspot model results suggest that increased risk heterogeneity decreases the probability of a large outbreak compared to the homogeneous case, a finding that confirms results from previous modeling analyses of superspreading events (2, 3). We define a large outbreak (or simply, an outbreak) as one in which at least 5% of the population becomes infected, and as we increase the relative contribution of hotspot to disease spread in our model, we project lower probability of an outbreak (Fig. 2—comparing across panels). Comparing scenarios with the same $R_{0}$ and proportion of hotspot spread, we find that populations with a lower mean risk-tolerance saw smaller probabilities of disease outbreak (Fig. 2—lines of different color diverge)—in these scenarios, risk is more heavily concentrated in a smaller portion of the population so the scenario is more heterogeneous. Surprisingly, we found that the *variance* of the distribution of risk tolerance across the population plays no role (Fig. 2—lines of different texture are indistinguishable).

### Outbreak probability driven by first-order factors: $R_{0}$ and mean risk-tolerance $\bar{\rho }$

To better understand predicted outbreak probabilities, we follow (2, 3) and compare the results of the hotspot model to a branching process approximation in which we compute the probability of disease extinction *τ*. In the Supplementary material, we show that


(1) $$\tau =\bar{\rho }e^{\left(\beta_{c}+\bar{\rho }\beta_{h})N(\tau -1\right)}+(1-\bar{\rho })e^{\beta_{c}N(\tau -1)}.$$

We can solve Eq. 1 implicitly or through use of the W Lambert function to yield predictions which match the results from the hsSIR agent-based model for values of $R_{0}\geq 1$ (Fig. 2).

What can we learn from the agreement between the branching process model and the agent-based model? The branching process model ignores two factors present in the full agent-based model: (i) the early accelerative effect in an outbreak as infected agents with higher risk-tolerance are infected first and (ii) the differences in risk distribution shapes beyond their mean. The agreement between the branching process model and the agent-based model suggests that these two factors only become significant later in an outbreak, at which point disease extinction has already become vanishingly unlikely.

### Hotspot spread increases outbreak sizes for small $R_{0}$ but decreases them for large $R_{0}$

**Fig. 3.**
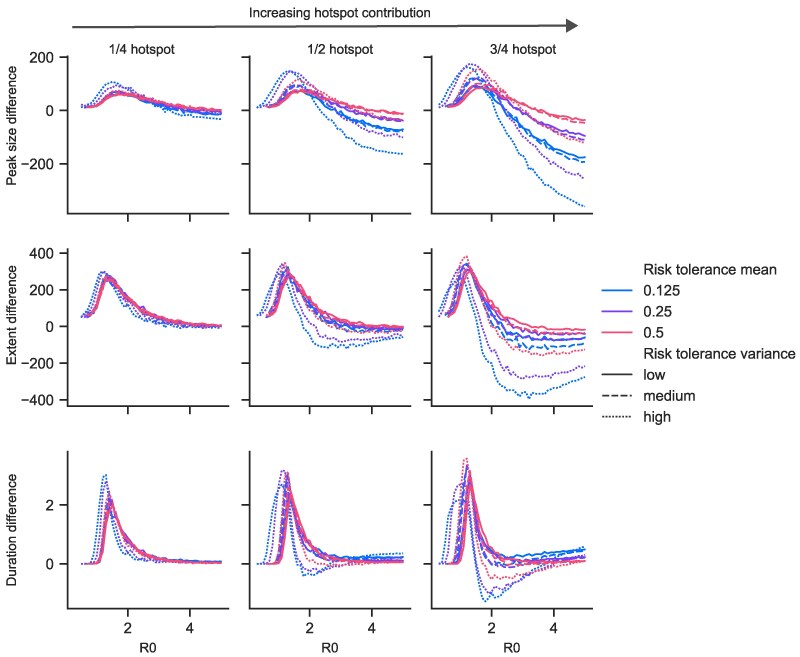
Effects of risk tolerance distribution on epidemic peak size, final size, and timing. (Top) Average peak size minus the peak size predicted by a homogeneous model with the same $R_{0}$. (Middle) Average final size minus the final size predicted by a homogeneous model with the same $R_{0}$. (Bottom) Average outbreak duration timing minus outbreak duration predicted by a homogeneous model with the same $R_{0}$. Peak size is the maximum number of infected agents at any given time during a simulation, final size is the total number of agents experiencing infection during a simulation, and outbreak duration is the time between the start of an outbreak (when at least 5% of the population is first infected) until the end of the outbreak (when the infected population first drops back below 5%), with time measured in recovery periods *D*. We limit to simulations in which an outbreak occurred (at least 5% of the population was infected). See Fig. S1 for peak size, final size, and timing shown without the corresponding homogeneous values subtracted, and Figs. S3–S7 for extended versions of this one.

Next, we investigate how elevated hotspot transmission affects the size and severity of the outbreak when one occurs. We consider peak size—the largest number of infected individuals at one time—and final size—the total number of individuals infected over the entire course of the outbreak. For very low $R_{0}$ (below 1.5), we see much larger outbreaks compared to the base homogeneous model (Fig. 3). Of course in the base model, an outbreak cannot happen at all with an $R_{0}$ below 1.0 (on average each individual infects 1 person)—but in the hotspot model transmission may persist within the highest risk individuals and outbreak may still occur. This phenomenon is highlighted in the inset of Fig. 2. The result is consistent with previous studies of high risk populations which result in heterogeneous $R_{0}$ that can be below 1.0 overall but greater than 1.0 in specific subpopulations; a result important for sexually transmitted infections (29, 30) and any disease where hosts have heterogeneous susceptibility or behavior (31). For moderate $R_{0}$ (from 1.5 to between 2.0–3.0), our model shows that outbreaks with significant hotspot transmission have a higher number of infections at their peak (higher peak size), but infect fewer individuals over their course (smaller final size) (Fig. 3). For high $R_{0}$ (above 2.0–3.0), outbreaks have both lower peak and smaller final size. These findings are consistently more pronounced for the risk tolerance distributions with (i) higher variance and (ii) lower mean risk-tolerance.

### Outbreak dynamics are driven by rise and fall of effective transmission rate

During an outbreak, the effective reproduction rate $R_{e}$ gives the expected number of new cases generated by an infected individual before they recover. In the homogeneous case,


(2) $$R_{e}=D\beta S.$$

On average an infected individual infects *β* proportion of the susceptible population *S* every day, and they remain infectious for *D* days. Thus, $R_{e}$ declines monotonically as the number of susceptible individuals decreases, the outbreak peaks when $R_{e}$ falls below 1, and cases begin to decline.

In the hotspot SIR model, we find an analogous expression for $R_{e}$ (see Materials and methods),


(3) $$R_{e}=D\beta_{c}S+D{\bar{\rho }}_{S}{\bar{\rho }}_{I}\beta_{h}S=D(\beta_{c}+{\bar{\rho }}_{S}{\bar{\rho }}_{I}\beta_{h})S.$$

This is similar to the homogeneous model but replaces the simple parameter *β* with the more complicated $\beta_{c}+{\bar{\rho }}_{S}{\bar{\rho }}_{I}\beta_{h}$, which depends on the mean risk-tolerance of the susceptible and infected populations ${\bar{\rho }}_{S}$ and ${\bar{\rho }}_{I}$. This dependency drives the dynamical differences between hotspot (hsSIR) and homogeneous standard SIR.

**Fig. 4.**
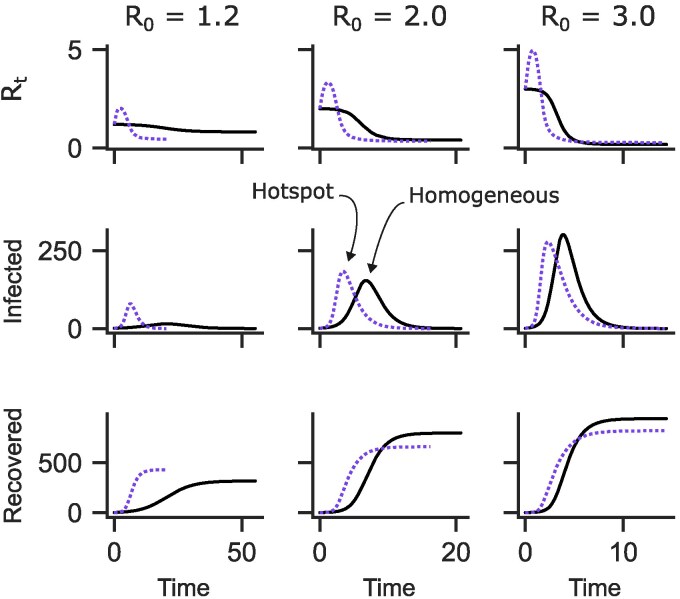
Effects of risk tolerance distribution on epidemic curves. Top panels show how $R_{t}$ in the hotspot model (dotted lines) rises and falls sharply, contrasted with the homogeneous model (black solid lines) in which $R_{t}$ declines monotonically. Middle and bottom panels show how this affects the peak and total number of infections, respectively. Time is measured in recovery periods, *D*. From left to right, with hotspot dynamics: low $R_{0}$ diseases peak higher and affect more people compared to homogeneous spread; medium $R_{0}$ diseases peak higher but affect fewer people; and high $R_{0}$ diseases peak lower and affect fewer people. Figures made with scenario of risk tolerance mean of 0.25, “high” risk tolerance distribution variance and 0.5 fraction of hotspot spread (middle right panel in Fig. 1B, dotted lines in the center column in Figs. 2 and 3). An extended version of this figure is available in Fig. S2.

Individuals who visit the hotspot are more likely to become infected early, pushing up the mean risk-tolerance of the infected population ${\bar{\rho }}_{I}$ and making higher-risk individuals even more likely to become infected. As these individuals with high risk-tolerance leave the susceptible population by becoming infected, ${\bar{\rho }}_{I}$ declines. At first, the susceptible population is large and the infected population is small, so this increases ${\bar{\rho }}_{I}$ more than it decreases ${\bar{\rho }}_{S}$ and creates a positive feedback loop in which $R_{e}$ increases rapidly. Eventually the susceptible population decreases in size, some number of high-risk individuals recover out of the infected population, and ${\bar{\rho }}_{I}$ itself starts to decline; at this point $R_{e}$ begins to decrease rapidly. As we saw in the previous section, this accelerates the rise and decline of the outbreak, exacerbating diseases with low $R_{0}$ while causing diseases with high $R_{0}$ to peak earlier and lower (Fig. 4).

### Variance of risk tolerance distribution increases the pace of outbreak dynamics

For large enough populations (i.e. as we increase *N* while decreasing $\beta_{h}$ and $\beta_{c}$ to hold $R_{0}$ constant), the behavior of an outbreak in the agent-based model is well described by a system of integrodifferential equations (see Supplementary information).

Given $R_{e}=D(\beta_{c}+{\bar{\rho }}_{S}{\bar{\rho }}_{I}\beta_{h})S$ (Eq. 3) for the basic reproduction number, understanding how ${\bar{\rho }}_{S}$ and ${\bar{\rho }}_{I}$ change over time is imperative to understanding the dynamics of hotspot disease spread. Using the integrodifferential system, we find


(4) $$\frac{d}{dt}{\bar{\rho }}_{S}=-\beta_{h}I{\bar{\rho }}_{I}\text{Var}(\rho_{S}),$$

and


(5) $$\frac{d}{dt}{\bar{\rho }}_{I}=S\left[\beta_{c}({\bar{\rho }}_{S}-{\bar{\rho }}_{I})+\beta_{h}{\bar{\rho }}_{S}{\bar{\rho }}_{I}\left(\left({\bar{\rho }}_{S}+\frac{\text{Var}(\rho_{S})}{{\bar{\rho }}_{S}}\right)-{\bar{\rho }}_{I}\right)\right].$$

In other words,

1. The mean risk-tolerance of the susceptible population (${\bar{\rho }}_{S}$) decreases at a rate proportional to force of infectious spread in the hotspot ($\beta_{h}$), the total infected population size (*I*), and the variance of the distribution of risk tolerance in the susceptible population ($\text{Var}(\rho_{S})$).
2. The mean risk-tolerance of the infected population (${\bar{\rho }}_{I}$) evolves towards a value between ${\bar{\rho }}_{S}$ and ${\bar{\rho }}_{S}(1+\text{Var}(\rho_{S})/({\bar{\rho }}_{S}{)}^{2})$.

Understanding the changes in ${\bar{\rho }}_{I}$ and ${\bar{\rho }}_{S}$ are important for projecting $R_{e}$ over time. We have found simple expressions for ${\bar{\rho }}_{I}$ and ${\bar{\rho }}_{S}$ featuring $\text{Var}(\rho_{s})$. This suggests that in higher variance risk-tolerance distributions—i.e. in scenarios in which a small number of people visit a hotspot frequently—${\bar{\rho }}_{S}$ decreases and ${\bar{\rho }}_{I}$ initially increases faster, heightening the accelerative outbreak dynamics that eventually lead to different outcomes than the homogeneous case.

### Disease recovery time has minimal effect on outbreak dynamics

Increasing disease recovery time *D* proportionately increases basic reproduction number $R_{0}$. However, we can vary *D while adjusting transmission rates $\beta_{h}$ and $\beta_{c}$ to keep $R_{0}$ unchanged*. If *D* is large, groups may gather in the hotspot, disperse, gather again, etc. all within one disease cycle. Conversely, when *D* is small, a single generation of the disease encounters a relatively fixed social network. Therefore, changing *D* in this way allows us to explore the impact of the relative rate at which fission–fusion dynamics occur.

However, when we vary *D* (while adjusting transmission rates to keep $R_{0}$ unchanged), we find little to no effect on the peak size, final size or overall rate of development outbreaks in the agent-based model (Supplementary information—Choice of *D* parameter section). We corroborate this finding by showing, when we develop the governing equations for $R_{e}$, ${\bar{\rho }}_{S}$ and ${\bar{\rho }}_{I}$ in the integrodifferential system model, that all terms containing disease recovery time *D* cancel out (Supplementary information—Integrodifferential system section) or can be parameterized away.

## Discussion

Epidemic models often include variation in individual susceptibility and transmissibility aiming to understand how heterogeneity affects transmission dynamics. The traditional modeling framework usually employs a simplified structure to handle risk distribution by dividing population in few (2–4) risk groups within which individuals have similar characteristics. For example, models of sexually transmitted diseases use behavioral categories such as number of sexual partners, frequency of sex acts and condom use to define high-risk individuals who are more likely to spread the disease and symmetrically more likely to become infected (32–39). Models of the acquisition and severity of airborne diseases, such as COVID, consider age, vaccination status, and mobility as individual-level traits (40–45). A previous theoretical analysis uses a structured infectious-disease model with demography, mass-action incidence, and an arbitrary number of risk classes to demonstrate that the basic reproduction number decreases as more risk classes are added even if the overall population risk remains the same (46).

Our model adds to this discussion of variation and heterogeneity in disease transmission in general, and risk structure in particular. We develop a minimal agent-based model that considers a simple dynamic for location-specific risk and risk-taking tendency. We complement this simulation model with analytic calculations based on simplifying assumptions to shed light on underlying mechanisms.

Our simulation model and analytic calculations predict three key findings for scenarios driven largely or primarily by hotspot spread. First, outbreaks are less likely to occur than in the homogeneous case. This is consistent with previous findings that enforce heterogeneity by specifying, for example, a distribution of secondary infections in branching processes (1, 47) or a contact distribution in network models (48, 49). However, in our model, heterogeneity in contact distributions is a mechanistic outcome of risk distribution and not directly enforced as an input. For example, high-degree nodes with many contacts, so-called hubs who are likely to lead to SSEs of type 2, can only take a single form in branching processes or network models. In our model, they can emerge because of individuals who regularly participate in hotspot encounters (SSEs of type 3) or because of individuals who do so simply at the wrong time (SSEs of type 4). In other words, hubs can be persistent in time or ephemeral and the heterogeneity of the contact distribution is a dynamical process.

Second, outbreaks that do occur are marked by a pronounced early acceleration and later deceleration of effective transmission rate. Epidemics with accelerating effective transmission rates have been observed before (50) but are typically explained by co-evolution models. In some cases, the contact structure changes in response to the epidemic (50), or multiple epidemics are interacting and feeding each other (51), or the efficiency of interventions like contact-tracing decreases as incidence grows (52). In our model, the effective transmission rate increases despite a static and very simple model because of correlations in risk patterns found in early infections.

Third, outbreaks with low $R_{0}$ have increased peak and final sizes while outbreaks with high $R_{0}$ have decreased peak and final sizes. This result is particularly important for epidemic forecasts and design of interventions as it suggests that $R_{0}$ alone provides incomplete information without knowledge of the underlying heterogeneity; a point that has been made repeatedly using network models during the COVID-19 pandemic (47). Here, we stress two important new caveats to this message. The underlying heterogeneity can have critical temporal patterns that will shift its impact on epidemic peak and final sizes. Are SSEs found around high-risk and consistently exposed individuals or opportunistic around sporadic hotspots? We are likely to always found a mixture of both types, but the precise mixture of risk found in these SSEs will affect forecasts. In particular, we can find large epidemics even with $R_{0}$ below 1, fueled by SSEs of type 3 when risk is sufficiently localized around high-risk individuals.

Our findings mostly refine our intuitions around the impact of temporal risk patterns during an epidemic but do also suggest some key applied considerations. The timing of interventions should consider which risk segment of the population is being targeted. Interventions targeted at high-risk individuals, such as closing or limiting capacity in bars, restaurants, or churches, may be significantly less useful by the time cases are high—their intended targets are more likely to have already been previously infected. Rather, interventions targeted at high-risk individuals and hotspots in particular should be preventative and applied to areas considered at risk *before* documented case numbers are high.

Attempts to quantify parameters such as $R_{0}$ or to predict case numbers over time and model the general development of an epidemic should consider the effect that risk structure can play. One should expect that outbreaks of diseases with high degree of superspreading, especially superspreading concentrated in high-risk individuals and/or moderated through location-based SSEs, will grow very fast in their early stages as if heading towards higher peaks and final sizes. Then later on, transmission rates will tend to level off.

Availability of cell phone data has made it possible to quantify in a very general way the frequency with which people visit specific locations, and we can use these results to think about which risk distributions (Fig. 1B) are most realistic. One influential recent result indicates that over broad ranges of time the number of people $N_{f}$ visiting a location with frequency *f* is proportional to $\frac{1}{f^{2}}$; i.e. that frequency of visitation is power law distributed with an exponent of 2 (53). This empirical pattern suggests that among distributions considered in Fig. 1B, those with the highest variance (dotted lines) are the most realistic for type 4 opportunistic superspreading events at bars, restaurants, churches, etc.

Our model makes a number of assumptions. We limit our focus to a fixed population in an isolated community. We allow only a single hotspot that is meant to represent multiple but well-mixed high-risk locations. Community spread is therefore homogeneous, and besides differing risk-tolerance values, individuals are identical in their individual infectiousness and susceptibility. We assume that individuals’ risk preferences do not vary with rising case numbers. Obviously none of these assumptions are realistic, but we view them as worthwhile to understand an idealized case that sheds light on the interplay between social fission–fusion dynamics and epidemic spread.

We believe that our model provides a framework for thinking about and analyzing outbreaks of disease with high location-specific effects in populations with quantifiable risk structures. We suggest a simple and unique mechanism that reinforces the message that diseases with superspreading are characterized by infrequent, large outbreaks. We also provide new findings suggesting that such outbreaks rise and fall faster than conventionally predicted. We hope our research can provide a useful starting point for future work that focuses on the role that variation in human movement and behavior plays in the spread of disease.

### Materials and methods

#### Basic and effective reproduction number

First, we define $R_{0}$ and $R_{e}$. The first infected agent has expected risk tolerance $\bar{\rho }$ and will recover in *D* time steps. Each time step, this agent goes to the hotspot with probability $\bar{\rho }$, encounters $\bar{\rho }N$ susceptible agents, and infects each of them with probability $\beta_{h}$. At the same time, the agent infects all other agents with probability $\beta_{c}$ each. This leads to the result that the expected value of the distribution of number of secondary cases is


(6) $$R_{0}=D({\bar{\rho }}^{2}\beta_{h}N)+D(\beta_{c}N)=D({\bar{\rho }}^{2}\beta_{h}+\beta_{c})N.$$

More generally, at any time we may ask the expected number of secondary infections caused by a single infected individual. An infected individual visiting the hotspot encounters an expected $\sum_{i\in S}\rho_{I}$ individuals and infects $\beta_{h}$ of them per time step. Averaging over susceptible individuals, this is $\beta_{h}\left(\sum_{\;j\in I}\rho_{j}\right)\left(\sum_{i\in S}\rho_{i}\right)/I$ or equivalently $\beta_{h}{\bar{\rho }}_{I}{\bar{\rho }}_{S}S$ infections. Simultaneously, an infected individual infects $\beta_{c}S$ individuals per time step through community spread, yielding that


(7) $$R_{e}=D({\bar{\rho }}_{I}{\bar{\rho }}_{S}\beta_{h}+\beta_{c})S.$$

### Model parameters

To investigate how disease dynamics and outcomes differ, we compare simulations with different levels of “hotspot” dynamics and different risk tolerance distributions while fixing initial basic reproduction number $R_{0}$.

We consider different shapes of the risk tolerance distribution P. For this, we use nine different parameterizations of a beta distribution to include Ps with low, medium and high variability; and low (1/8), medium (1/4), and high (1/2) mean. These are illustrated in Fig. 1B.

We use the value $f_{h}$, the fraction of $R_{0}$ due to hotspot spread, to select values of $\beta_{h}$ and $\beta_{c}$ that will produce a specified contribution of hotspot spread to $R_{0}$.

Letting $f_{h}$ be the fraction of $R_{0}$ due to hotspot spread,


(8) $$f_{h}=\frac{D{\bar{\rho }}^{2}\beta_{h}N}{D({\bar{\rho }}^{2}\beta_{h}+\beta_{c})N}=\frac{D{\bar{\rho }}^{2}\beta_{h}N}{R_{0}},$$

and we use the value of $f_{h}$ to link $\beta_{h}$ and $\beta_{h}$ to $R_{0}$. Rearranging Eq. 8, we have


(9) $$\beta_{h}=\frac{\;f_{h}R_{0}}{ND{\bar{\rho }}^{2}},$$

and


(10) $$\beta_{c}=\frac{(1-f_{h})R_{0}}{ND}.$$

We consider values of $f_{h}\in \{1/4,1/2,3/4\}$ and use the previous equations to set values of parameters $\beta_{h}$ and $\beta_{h}$ when running simulations for $R_{0}$ ranging from 0 to 5.

We compare the outcomes of the outbreaks to those with no risk-taking but identical $R_{0}$ (i.e, where $f_{h}=\beta_{h}=0$). See the Supplementary information for more details.

### Other models

To aid in investigating the dynamics of the model in certain cases, we consider and analytically analyze a branching process model and an integrodifferential system model. We describe these models in detail in the Supplementary information.

## Supplementary Material

pgaf299_Supplementary_Data

## Data Availability

All code and data are available at GitHub, https://github.com/brendanwallace/hotspot.
