# Supplementary material for: Hotspot model shows how location-based superspreading accelerates and reshapes epidemics
Source: PNAS Nexus. 2025 Sep 22;4(9):pgaf299. doi: 10.1093/pnasnexus/pgaf299 (PMC12481239; doi:10.1093/pnasnexus/pgaf299)
Supplement: pgaf299_Supplementary_Data [file pgaf299_supplementary_data.pdf]

Supporting Information Text

Contents

1 Branching process model & disease extinction 1
A Homogeneous case 1
B Hotspot case 2

2 Integrodifferential equations model 2
A Model derivation and introduction 2
B Moment relation Lemma 3
C Basic and effective reproduction numbers 4
D Mean risk tolerance 4

3 Model parameters 6
A Determining model parameters for simulations 6
B Recovery time 7

1. Branching process model & disease extinction

A newly invading disease in a large population grows approximately exponentially as $(R_0)^t$ where R_0 is the basic reproduction rate of that disease and t is a measure of time, as each infected agent infects R_0 others before recovering. However, in real life as in our stochastic agent-based model, there is some probability of the invading disease failing to cause a large outbreak. Here we derive an approximate expression for the probability of no large outbreak occuring, called "extinction" of the disease and denoted τ, using a branching process approximation.

We make the following assumptions:

1. Branching process assumption

We treat all newly infected individuals as the first and only infected individuals in new simulations with N susceptible agents, rather than considering the decreasing pool of susceptible individuals in the simulation as an outbreak progresses.

2. Unbiased risk tolerance assumption When a new individual is infected, we treat their risk tolerance as drawn randomly from the population distribution of risk tolerances, rather than being drawn with bias towards higher risk tolerances due to the increased likelihood of infection in such individuals.

3. Large N, small β assumption

We take N to be very large and β_c to be very small, with $N\beta_c = R_0$ constant. (This is not strictly necessary, but allows for a few nice simplifications of the results.)

A. Homogeneous case. Suppose an infected individual is introduced to a susceptible population of N individuals. For simplicity, assume $D = 1$ (see section B for other choices of D). We begin with the homogeneous case, $\beta_h = 0$, $\rho_i = 0$, and therefore $R_0 = \beta_c N$.

Let random variables X_i represent the number of infections caused by the ith infection before that infected agent recovers. X_0, the infections caused by the initial infection, is binomial with

$$P(X_0 = n) = \binom{N}{n} \beta_c^n (1 - \beta_c)^{N-n}. \quad [1]$$

Now with (**1. Branching process assumption**), we treat all X_i as i.i.d. binomial with $p = \beta_c$ and $N = N$, so that with τ being the probability of disease extinction,

$$\tau = \sum_{n=0}^{N} P(X_0 = n)\tau^n = (1 + \beta_c(\tau - 1))^N. \quad [2]$$

Since with $P(X = 0)$, the first infection causes zero infections and the disease goes extinct, but with probability $P(X = n)$, n identical processes begin which *all* go extinct with probability τ^n. The closed form expression is the probability generating function (PGF) for the binomial distribution, which we are justified in using under the assumption that all X_i are i.i.d. binomial.

And with (**3. Large N, small β assumption**) we consider the limit,

$$\tau \approx \lim_{N\to\infty} \left(1 + \frac{\beta_c N(\tau - 1)}{N}\right)^N = e^{\beta_c N(\tau-1)} = e^{R_0(\tau-1)}. \quad [3]$$

Alternatively, taking the limit of $N \to \infty$ while holding $\beta_c N$ constant we replace the binomial X_i with a Poisson variable and use the probability generating function for a Poisson variable to arrive at the equivalent result.

B. Hotspot case. Next we consider the full hotspot case.

Let ρ_0 be the risk tolerance of an initial infected agent introduced to a population of N susceptible individuals, with β_c, $\beta_h > 0$ and $D = 1$, and look for the probability of extinction in a similar branching process. However, we must be careful here in noting that τ may depend on which initial agent is randomly drawn so we write $\tau(\rho_0)$, and define $\tau = E[\tau(\rho_0)]$.

The first infected agent visits the hotspot during their infection with probability ρ_0. If they do visit the hotspot, they infect other agent j through hotspot spread with probability:

$$\int_0^1 \rho_j \beta_h d\rho_j = \bar{\rho}\beta_h \tag{4}$$

Combined with community spread, this is $\beta_c + (1 - \beta_c)(\bar{\rho}\beta_h)$, or taking $\beta_c, \beta_h << 1$ and ignoring terms of $o(\beta^2)$, just $\beta_c + \bar{\rho}\beta_h$.

With probability ρ_0 the first agent does not visit the hotspot, and infects other agents with conditional probability β_c.

This leads to, (with p_0 given),

$$P(X_0 = n) = \rho_0 \binom{N}{n} ((\beta_c + \rho_j\beta_h)^n (1 - (\beta_c + \rho_j\beta_h))^{N-n}) + (1 - \rho_0) \binom{N}{n} \beta_c^n (1 - \beta_c)^{N-n}. \tag{5}$$

It is important to note that individual 0 either visits the hotspot during their infectious period or does not, so ρ_0 is realized only once not for each interaction. Conversely, all other individuals have iid probabilities $\bar{\rho}$ of visiting the hotspot over this period after considering first their draws from the risk tolerance distribution and then the realizations of their risk taking behavior.

In expectation, we may replace ρ_0 above with $\bar{\rho}$. Then, making use of the **1. Branching process assumption** and **2. Unbiased risk tolerance assumption**, we have $X_i = X_0$. So we can now calculate the branching process extinction probability,

$$\begin{aligned}
\tau &= \sum_{n=0}^{N} P(X_0)\tau^n \\
&= \sum_{n=0}^{N} \left(\bar{\rho} \binom{N}{n} (\beta_c + \bar{\rho}\beta_h)^n (1 - (\beta_c + \bar{\rho}\beta_h))^{N-n} + (1 - \bar{\rho}) \binom{N}{n} \beta_c^n (1 - \beta_c)^{N-n} \right) \tau^n \\
&= \bar{\rho} \sum_{n=0}^{N} \left(\binom{N}{n} (\beta_c + \bar{\rho}\beta_h)^n (1 - (\beta_c + \bar{\rho}\beta_h))^{N-n} \right) \tau^n + (1 - \bar{\rho}) \sum_{n=0}^{N} \left(\binom{N}{n} \beta_c^n (1 - \beta_c)^{N-n} \right) \tau^n \\
&= \bar{\rho}(1 + (\beta_c + \bar{\rho}\beta_h)(\tau - 1))^N + (1 + \bar{\rho})(1 - \beta_c(\tau - 1))^N
\end{aligned} \tag{6}$$

Similar to the homogeneous case, we use (**3. Large N, small β assumption**) to consider these terms as N increases while R_0 remains fixed.

$$\lim_{N\to\infty} (1 + (\beta_c + \bar{\rho}\beta_h)(\tau - 1))^N = \lim_{N\to\infty} (1 + \frac{(\beta_c + \bar{\rho}\beta_h)N(\tau - 1)}{N})^N = e^{(\beta_c + \bar{\rho}\beta_h)N(\tau - 1)}, \tag{7}$$

and

$$\lim_{N\to\infty} (1 + \beta_c(\tau - 1))^N = \lim_{N\to\infty} (1 + \frac{\beta_c N(\tau - 1)}{N})^N = e^{\beta_c N(\tau - 1)}. \tag{8}$$

This leads to

$$\tau = \bar{\rho}e^{(\beta_c + \bar{\rho}\beta_h)N(\tau - 1)} + (1 - \bar{\rho})e^{\beta_c N(\tau - 1)}. \tag{9}$$

2. Integrodifferential equations model

A. Model derivation and introduction. As N grows large, instead of considering individuals with individual risk tolerance values ρ_i we consider the population *density* as a function of ρ, where $S(\rho)$, $I(\rho)$, and $R(\rho)$ are the susceptible, infected, and recovered population densities with risk tolerance value ρ, respectively.

Then, we consider how the population density at a given value of ρ changes over time. So we look for $\frac{\partial S(\rho)}{\partial t}$, and $\frac{\partial I(\rho)}{\partial t}$ and $\frac{\partial R(\rho)}{\partial t}$, where S, I, and R are functions of both time t and risk tolerance ρ so we use partial derivatives.

Starting with $\frac{\partial S(\rho)}{\partial t}$, we must consider community spread and hotspot spread.

The entire infected population acts through community spread to infect the susceptible population equally at all values of ρ, so that community spread at risk value ρ is equal to

$$\beta_c S(\rho) \int_0^1 I(\tilde{\rho}) d\tilde{\rho}, \tag{10}$$

where $\tilde{\rho}$ is the risk tolerance of integration over the infected population.

Simultaneously, fraction ρ of the susceptible population *with risk tolerance* ρ visits the hotspot and is infected by the proportion of all densities of I which also visit the hotspot. This leads to hotspot infections equal to

$$\beta_h \rho S(\rho) \int_0^1 \tilde{\rho} I(\tilde{\rho}) d\tilde{\rho}. \tag{11}$$

Therefore, we have

$$\frac{\partial S(\rho)}{\partial t} = -\beta_c S(\rho) \int_0^1 I(\tilde{\rho}) d\tilde{\rho} - \beta_h \rho S(\rho) \int_0^1 \tilde{\rho} I(\tilde{\rho}) d\tilde{\rho}, \tag{12}$$

and,

$$\frac{\partial I(\rho)}{\partial t} = \beta_c S(\rho) \int_0^1 I(\tilde{\rho}) d\tilde{\rho} + \beta_h S(\rho) \rho \int_0^1 \tilde{\rho} I(\tilde{\rho}) d\tilde{\rho} - \frac{1}{D} I(\rho), \tag{13}$$

with fraction $1/D$ of the infected population recovering.

For convenience, we introduce the following short-hands for the "moments" of $I(\rho)$ and $S(\rho)$,

$$S := \int_0^1 S(\rho) d\rho \tag{14}$$

and

$$I := \int_0^1 I(\rho) d\rho \tag{15}$$

are the zeroth moments.

$$\overset{(1)}{S} := \int_0^1 \rho S(\rho) d\rho \tag{16}$$

and

$$\overset{(1)}{I} := \int_0^1 \rho I(\rho) d\rho \tag{17}$$

are the first moments.

And in general,

$$\overset{(n)}{S} := \int_0^1 \rho^n S(\rho) d\rho \tag{18}$$

and

$$\overset{(n)}{I} := \int_0^1 \rho^n I(\rho) d\rho \tag{19}$$

are the nth moments.

Written with these short-hands,

$$\frac{\partial S(\rho)}{\partial t} = -\beta_c S(\rho) I - \beta_h \rho S(\rho) \overset{(1)}{I}, \tag{20}$$

and

$$\frac{\partial I(\rho)}{\partial t} = \beta_c S(\rho) I + \beta_h \rho S(\rho) \overset{(1)}{I} - \frac{1}{D} I(p) \tag{21}$$

B. Moment relation Lemma. As a lemma we'll show that

$$\frac{d \overset{(n)}{S}}{dt} = -\beta_c \overset{(n)}{S} I - \beta_h \overset{(n+1)}{S} \overset{(1)}{I}, \tag{22}$$

and

$$\frac{d \overset{(n)}{I}}{dt} = \beta_c \overset{(n)}{S} I + \beta_h \overset{(n+1)}{S} \overset{(1)}{I} - \frac{1}{D} \overset{(n)}{I}. \tag{23}$$

(Recall that $\overset{(n)}{S}$ and $\overset{(n)}{I}$ are not functions of ρ because they are the results of integrations performed over ρ)

We prove this Lemma by differentiating, such that

$$
\begin{aligned}
\frac{d\overset{(n)}{S}}{dt} &= \frac{d}{dt}\left(\int_0^1 S(\rho)\rho^n d\rho\right) \\
&= \int_0^1 \frac{\partial S(\rho)}{\partial t}\rho^n d\rho \\
&= \int_0^1 (-\beta_c S(\rho) I - \beta_h \rho S(\rho)\overset{(1)}{I})\rho^n d\rho \\
&= -\beta_c(\int_0^1 S(\rho)\rho^n d\rho) I - \beta_h(\int_0^1 S(\rho)\rho^{n+1} d\rho)\overset{(1)}{I} \\
&= -\beta_c \overset{(n)}{S} I - \beta_h \overset{(n+1)}{S}\overset{(1)}{I}
\end{aligned}
\qquad [24]
$$

. Similarly,

$$
\begin{aligned}
\frac{d\overset{(n)}{I}}{dt} &= \frac{d}{dt}\left(\int_0^1 I(\rho)\rho^n d\rho\right) \\
&= \int_0^1 \frac{\partial I(\rho)}{\partial t}\rho^n d\rho \\
&= \int_0^1 (\beta_c S(\rho) I + \beta_h \rho S(\rho)\overset{(1)}{I} - \frac{1}{D} I(\rho))\rho^n d\rho \\
&= \beta_c(\int_0^1 S(\rho)\rho^n d\rho) I + \beta_h(\int_0^1 S(\rho)\rho^{n+1} d\rho)\overset{(1)}{I} - \frac{1}{D}(\int_0^1 I(\rho)\rho^n d\rho) \\
&= \beta_c \overset{(n)}{S} I + \beta_h \overset{(n+1)}{S}\overset{(1)}{I} - \frac{1}{D}\overset{(n)}{I}.
\end{aligned}
\qquad [25]
$$

C. Basic and effective reproduction numbers. R_e is the instantaneous rate of new infections per infection, so,

$$
R_e = -\frac{dS}{dt}\frac{D}{I}. \qquad [26]
$$

Using the lemma above,

$$
\begin{aligned}
R_e &= (\beta_c S I + \beta_h \overset{(1)}{S}\overset{(1)}{I})\frac{D}{I} \\
&= DS(\beta_c + \beta_h \frac{\overset{(1)}{S}}{S}\frac{\overset{(1)}{I}}{I})
\end{aligned}
\qquad [27]
$$

Recall that $\overset{(1)}{S} = \int_0^1 \rho S(\rho) d\rho$ and $S = \int_0^1 S(\rho) d\rho$. Therefore, the term $\frac{\overset{(1)}{S}}{S}$ refers to the mean risk tolerance value in the susceptible population. And similarly, $\frac{\overset{(1)}{I}}{I}$ is the mean risk tolerance in the infected population. We denote these as $\bar{\rho}_S$ and $\bar{\rho}_I$ respectively, so that,

$$
R_e = DS(\beta_c + \beta_h \bar{\rho}_S \bar{\rho}_I). \qquad [28]
$$

To find R_0 we note that all agents start susceptible, so $N = S$ and $\bar{\rho}_S = \bar{\rho}$. We initialize ϵ of the population in proportion to $\bar{\rho}$ so that $\bar{\rho}_I = \bar{\rho}$ as well. Then,

$$
R_0 = DN(\beta_c + \bar{\rho}^2 \beta_h). \qquad [29]
$$

D. Mean risk tolerance. Recall that $\bar{\rho}_S = \overset{(1)}{S}/S$. Differentiate using the quotient rule, then substitute expressions from the lemma for $\frac{d}{dt}(\overset{(1)}{S})$ and $\frac{d}{dt}(S)$.

$$\begin{aligned}
\frac{d}{dt}\bar{\rho}_S &= \frac{d}{dt}\frac{\overset{(1)}{S}}{S} \\
&= \frac{\frac{d}{dt}(\overset{(1)}{S})S - \overset{(1)}{S}\frac{d}{dt}(S)}{S^2} \\
&= \frac{1}{S}(-\beta_c \overset{(1)}{S} I - \beta_h \overset{(2)}{S}\overset{(1)}{I}) - \frac{\overset{(1)}{S}}{S^2}(-\beta_c S I - \beta_h \overset{(1)}{S}\overset{(1)}{I}) \\
&= -\beta_c \frac{\overset{(1)}{S}}{S} I - \beta_h \frac{\overset{(2)}{S}}{S}\overset{(1)}{I} + \beta_c \frac{\overset{(1)}{S}}{S} I + \beta_h (\frac{\overset{(1)}{S}}{S})^2 \\
&= -\beta_h \frac{\overset{(2)}{S}}{S}\overset{(1)}{I} + \beta_h (\frac{\overset{(1)}{S}}{S})^2 \\
&= -\beta_h \overset{(1)}{I}(\frac{\overset{(2)}{S}}{S} - (\frac{\overset{(1)}{S}}{S})^2).
\end{aligned} \tag{30}$$

Here, notice $\overset{(2)}{S}$ sums ρ^2 over $[0,1]$ and so $\frac{\overset{(2)}{S}}{S}$ we can write as $E[\rho_S^2]$. And similarly $\frac{\overset{(1)}{S}}{\bar{S}} = E[\rho_S]$ so

$$\frac{\overset{(2)}{S}}{S} - (\frac{\overset{(1)}{S}}{S})^2 = E[\rho_S^2] - E[\rho_S]^2. \tag{31}$$

which is the variance of the risk tolerance of the susceptible population $Var(\rho_S)$. This leads to

$$\frac{d}{dt}\bar{\rho}_S = -\beta_h \overset{(1)}{I}\,\mathrm{Var}(\rho_s), \tag{32}$$

or

$$\frac{d}{dt}\bar{\rho}_S = -\beta_h \overset{(1)}{I}\bar{\rho}_I \mathrm{Var}(\rho_s). \tag{33}$$

Similarly, start with $\bar{\rho}_I = \overset{(1)}{I}/I$ and differentiate with the quotient rule and then substitute in from the moment equations for $\frac{d}{dt}(\overset{(1)}{S})$ and $\frac{d}{dt}(\bar{I})$.

$$\begin{aligned}
\frac{d}{dt}\bar{\rho}_I &= \frac{d}{dt}(\frac{\overset{(1)}{I}}{I}) \\
&= \frac{\frac{d}{dt}(\overset{(1)}{I})I - \overset{(1)}{I}\frac{d}{dt}(I)}{I^2} \\
&= [(\beta_c \overset{(1)}{S} I + \beta_h \overset{(2)}{S}\overset{(1)}{I} - \frac{1}{D}\overset{(1)}{I})I - \overset{(1)}{I}(\beta_c S I + \beta_h \overset{(1)}{S}\overset{(1)}{I} - \frac{1}{D}I)]/I^2 \\
&= [(\beta_c(\overset{(1)}{S} I^2 - S\overset{(1)}{I} I) + \beta_h(\overset{(2)}{S}\overset{(1)}{I} I - \overset{(1)}{S}\overset{(1)}{I}^2) + \frac{1}{D}(\overset{(1)}{I} I - \overset{(1)}{I} I)]/I^2.
\end{aligned} \tag{34}$$

Here, remarkably all terms with D cancel out. We continue by dividing through by I^2 while factoring out S. Then we rearrange factors in the β_h term.

$$\begin{aligned}
&= S[\beta_c((\overset{(1)}{S}/S) - (\overset{(1)}{I}/I)) + \beta_h((\overset{(2)}{S}/S)(\overset{(1)}{I}/I) - (\overset{(1)}{S}/S)(\overset{(1)}{I}/I)^2)] \\
&= S[\beta_c((\overset{(1)}{S}/S) - (\overset{(1)}{I}/I)) + \beta_h(\overset{(1)}{S}/S)((\overset{(2)}{S}/\overset{(1)}{S})(\overset{(1)}{I}/I) - (\overset{(1)}{I}/I)^2)]
\end{aligned} \tag{35}$$

Next substitute $\overset{(1)}{I}/I = \bar{\rho}_I$ and $\overset{(1)}{S}/\bar{S} = \bar{\rho}_S$

$$\begin{aligned}
&= \bar{S}[\beta_c(\bar{\rho}_S - \bar{\rho}_I) + \beta_h \bar{\rho}_S((\overset{(2)}{S}/\overset{(1)}{S})\bar{\rho}_I - \bar{\rho}_I^2)] \\
&= \bar{S}[\beta_c(\bar{\rho}_S - \bar{\rho}_I) + \beta_h \bar{\rho}_I \bar{\rho}_S((\overset{(2)}{S}/\overset{(1)}{S}) - \bar{\rho}_I)]
\end{aligned} \tag{36}$$

Finally,

$$(\overset{(2)}{S}/\overset{(1)}{S}) = \frac{\overset{(2)}{S}/S}{\overset{(1)}{S}/S} = \frac{E[\rho_S^2]}{\bar{\rho}_S} = \frac{\text{Var}(\rho_S) + E[\rho_S]^2}{\bar{\rho}_S} = \frac{\text{Var}(\rho_S)}{\bar{\rho}_S} + \frac{\bar{\rho}_S^2}{\bar{\rho}_S} = \frac{\text{Var}(\rho_S)}{\bar{\rho}_S} + \bar{\rho}_S \quad [37]$$

And we have that

$$\frac{d}{dt}\bar{\rho}_I = S\left[\beta_c(\bar{\rho}_S - \bar{\rho}_I) + \beta_h\bar{\rho}_S\bar{\rho}_I((\bar{\rho}_S + \frac{\text{Var}(\bar{\rho}_S)}{\bar{\rho}_S}) - \bar{\rho}_I))\right]. \quad [38]$$

3. Model parameters

A. Determining model parameters for simulations. The hotspot SIR model has only the following *model parameters*: recovery time D, population size N, infection rates β_c and β_h, and risk tolerance values ρ_i (drawn from distribution P).

As discussed in the main text, we set these *model parameters* for a given simulation by choosing some of the parameters, fixing certain *computed quantities*, and then allowing β_c and β_h to vary. Specifically, we follow the proceeding steps.

- Choose recovery time D. See section B for more details.
- Choose population size N. This is 1,000 for all figures in the main text.
- Choose mean risk tolerance $\bar{\rho}$ as one of 0.125 (low mean), 0.25 (medium mean), 0.5 (high mean). Note that *low* risk tolerance *mean* equates to *high* risk tolerance *concentration*.
- Choose variance factor one of 2.0 (low variance), 1.0 (medium variance) or 0.1 (high variance).
- Set the risk tolerance distribution P as a Beta(A, B) distribution with $A =$ variance factor. For $E[\text{Beta(A, B)}] = \bar{\rho}$ as given, we must set

$$B = \frac{(\text{variance factor})(1-\bar{\rho})}{\bar{\rho}}. \quad [39]$$

- Choose the contribution of hotspot spread f_h (portion of R_0 due to spread in hotspots) as 0.25, 0.5, or 0.75 (or 0, for the homogeneous case).
- Choose R_0. We show values from 0 to 5 for figures in the main text.
- For R_0 and hotspot spread as chosen, we set infection rates,

$$\beta_c = (1-f_h)\frac{R_0}{DN}, \quad [40]$$

  and

$$\beta_h = f_h\frac{R_0}{DN\bar{\rho}^2}. \quad [41]$$

This procedure is summarized visually in Table S1, and numerical values for an example set of R_0 are shown in Table S2.

Value	Description	Value(s)
D	Recovery time	$[1, 8]$
N	Population size	1000
$\bar{\rho}$	Risk tolerance mean	$\{0.125, 0.25, 0.5\}$
A	Beta distribution parameter	$\{2.0, 1.0, 0.1\}$
B	Beta distribution parameter	$\frac{(A)(1-\bar{\rho})}{\bar{\rho}}$
P	Risk tolerance distribution	Beta(A, B)
f_h	Hotspot fraction	$\{0, 0.25, 0.5, 0.75\}$
R_0	Basic transmission number	$[0.0, 5.0]$
β_c	Community transmission rate	$\beta_c = (1-f_h)\frac{R_0}{DN}$
β_h	Hotspot transmission rate	$\beta_h = f_h\frac{R_0}{DN\bar{\rho}^2}$

Table S1. Visual summary of the model parameter selection procedure.

Risk mean $\bar{\rho}$	Risk variance Var(ρ)	Beta parameter A	Beta parameter B	R_0	Hotspot fraction	β_c	β_h
0.125	low	2	14	2	0.25	0.0015	0.032
0.125	low	2	14	2	0.5	0.001	0.064
0.125	low	2	14	2	0.75	0.0005	0.096
0.125	low	2	14	4	0.25	0.003	0.064
0.125	low	2	14	4	0.5	0.002	0.128
0.125	low	2	14	4	0.75	0.001	0.192
0.125	medium	1	7	2	0.25	0.0015	0.032
0.125	medium	1	7	2	0.5	0.001	0.064
0.125	medium	1	7	2	0.75	0.0005	0.096
0.125	medium	1	7	4	0.25	0.003	0.064
0.125	medium	1	7	4	0.5	0.002	0.128
0.125	medium	1	7	4	0.75	0.001	0.192
0.125	high	0.1	0.7	2	0.25	0.0015	0.032
0.125	high	0.1	0.7	2	0.5	0.001	0.064
0.125	high	0.1	0.7	2	0.75	0.0005	0.096
0.125	high	0.1	0.7	4	0.25	0.003	0.064
0.125	high	0.1	0.7	4	0.5	0.002	0.128
0.125	high	0.1	0.7	4	0.75	0.001	0.192
0.25	low	2	6	2	0.25	0.0015	0.008
0.25	low	2	6	2	0.5	0.001	0.016
0.25	low	2	6	2	0.75	0.0005	0.024
0.25	low	2	6	4	0.25	0.003	0.016
0.25	low	2	6	4	0.5	0.002	0.032
0.25	low	2	6	4	0.75	0.001	0.048
0.25	medium	1	3	2	0.25	0.0015	0.008
0.25	medium	1	3	2	0.5	0.001	0.016
0.25	medium	1	3	2	0.75	0.0005	0.024
0.25	medium	1	3	4	0.25	0.003	0.016
0.25	medium	1	3	4	0.5	0.002	0.032
0.25	medium	1	3	4	0.75	0.001	0.048
0.25	high	0.1	0.3	2	0.25	0.0015	0.008
0.25	high	0.1	0.3	2	0.5	0.001	0.016
0.25	high	0.1	0.3	2	0.75	0.0005	0.024
0.25	high	0.1	0.3	4	0.25	0.003	0.016
0.25	high	0.1	0.3	4	0.5	0.002	0.032
0.25	high	0.1	0.3	4	0.75	0.001	0.048
0.5	low	2	2	2	0.25	0.0015	0.002
0.5	low	2	2	2	0.5	0.001	0.004
0.5	low	2	2	2	0.75	0.0005	0.006
0.5	low	2	2	4	0.25	0.003	0.004
0.5	low	2	2	4	0.5	0.002	0.008
0.5	low	2	2	4	0.75	0.001	0.012
0.5	medium	1	1	2	0.25	0.0015	0.002
0.5	medium	1	1	2	0.5	0.001	0.004
0.5	medium	1	1	2	0.75	0.0005	0.006
0.5	medium	1	1	4	0.25	0.003	0.004
0.5	medium	1	1	4	0.5	0.002	0.008
0.5	medium	1	1	4	0.75	0.001	0.012
0.5	high	0.1	0.1	2	0.25	0.0015	0.002
0.5	high	0.1	0.1	2	0.5	0.001	0.004
0.5	high	0.1	0.1	2	0.75	0.0005	0.006
0.5	high	0.1	0.1	4	0.25	0.003	0.004
0.5	high	0.1	0.1	4	0.5	0.002	0.008
0.5	high	0.1	0.1	4	0.75	0.001	0.012

Table S2. Model parameters and computed quantities shown for an example subset of R_0 values.

B. Recovery time. We vary recovery time D to observe the affects on our model results by adjusting β_h and β_c such that we keep R_0 unchanged (via equation $R_0 = D(\bar{\rho}^2\beta_h + \beta_c)N$ – see section A).

We generally do not consider $D < 1$. *

*If $D < 1$ then multiple disease generations can play out before there's a change in which people are gathered at the hot spot, and in the limiting case $D << 1$ the disease outbreak occurs on a fully static weighted network. In small D scenarios, the risk tolerance distribution across the population matters less than the *realization* of risk taking that occurred in a particular simulation or scenario and therefore our hotspot model is of limited utility.

To keep R_0 fixed while increasing D, we proportionately decrease individual infection rates, and the outbreak evolves much more slowly in overall terms. However, this effect is obvious and somewhat trivial, so to make more meaningful comparisons we consider how varying D changes the dynamics and timing of outbreaks *relative to a timescale normalized to* D.

We find that D has no significant effect the peak and final number of infections during an outbreak (see Fig. S4 and Fig. S5). Primarily we see a smoothing of the resulting curves as D increases – due to removal of the artifact of the periodicity. Similarly, epidemic curves *when the timescale is normalized with respect to* D are unaffected. We conclude that increasing D pushes the agent-based model towards the more smooth integrodifferential equation system model.

Consistent with this interpretation, we find that increasing D (while keeping R_0 unchanged) slightly decreases the probability of disease extinction (see: Fig. S3). This is to be expected: as we increase D, the expected number of secondary infections generated by the first infected individual (R_0) remains the same, but the variance of the number of secondary infections decreases slightly, while remaining higher than in the homogeneous case.

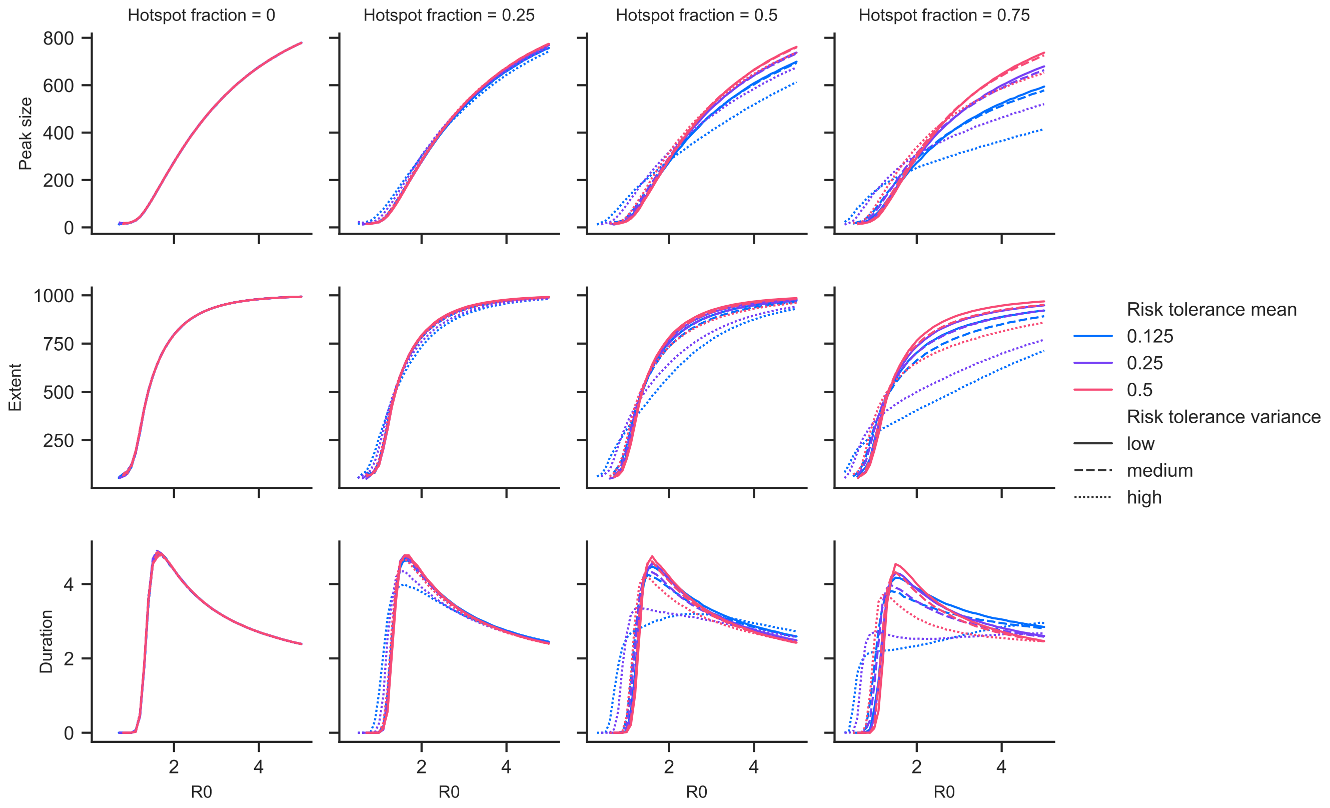

Fig. S1. Effects of risk tolerance distribution on epidemic peak, final size and timing. Colored lines show average outbreak peak size (Top), final size (Middle), and duration (Bottom). Columns show different proportions of hotspot contribution; in the leftmost column hotspot fraction of 0 shows the homogeneous model.

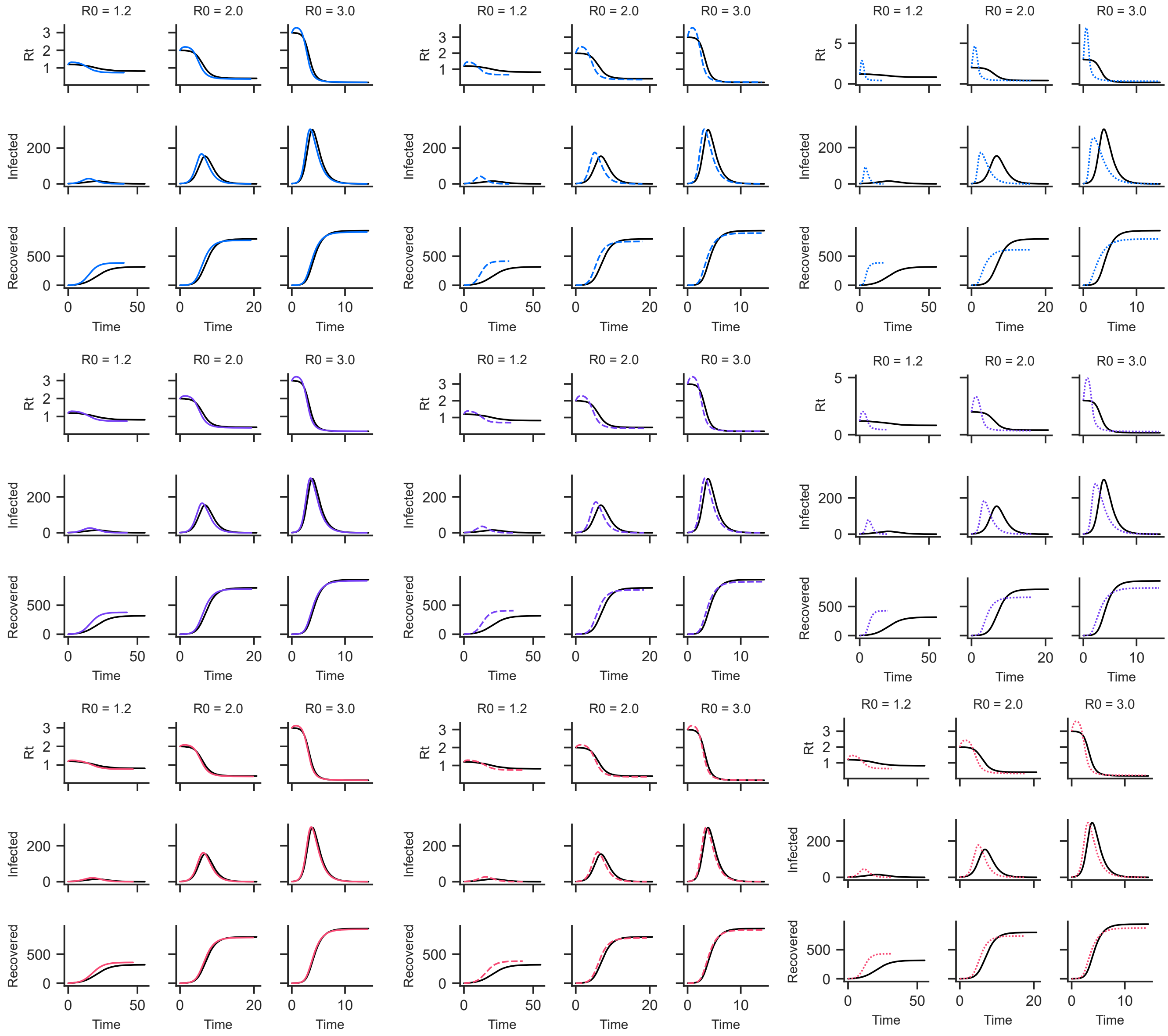

Fig. S2. Effects of risk tolerance distribution on epidemic curves - extended. Main text Figure 4 is replicated for each of the 9 scenarios shown in Fig. 1B. In each pane, the top panels show R_t in the hotspot model (colored lines) rises contrasted with the homogeneous model (black solid lines). Middle and bottom panels show how this affects the peak and total number of infections respectively.

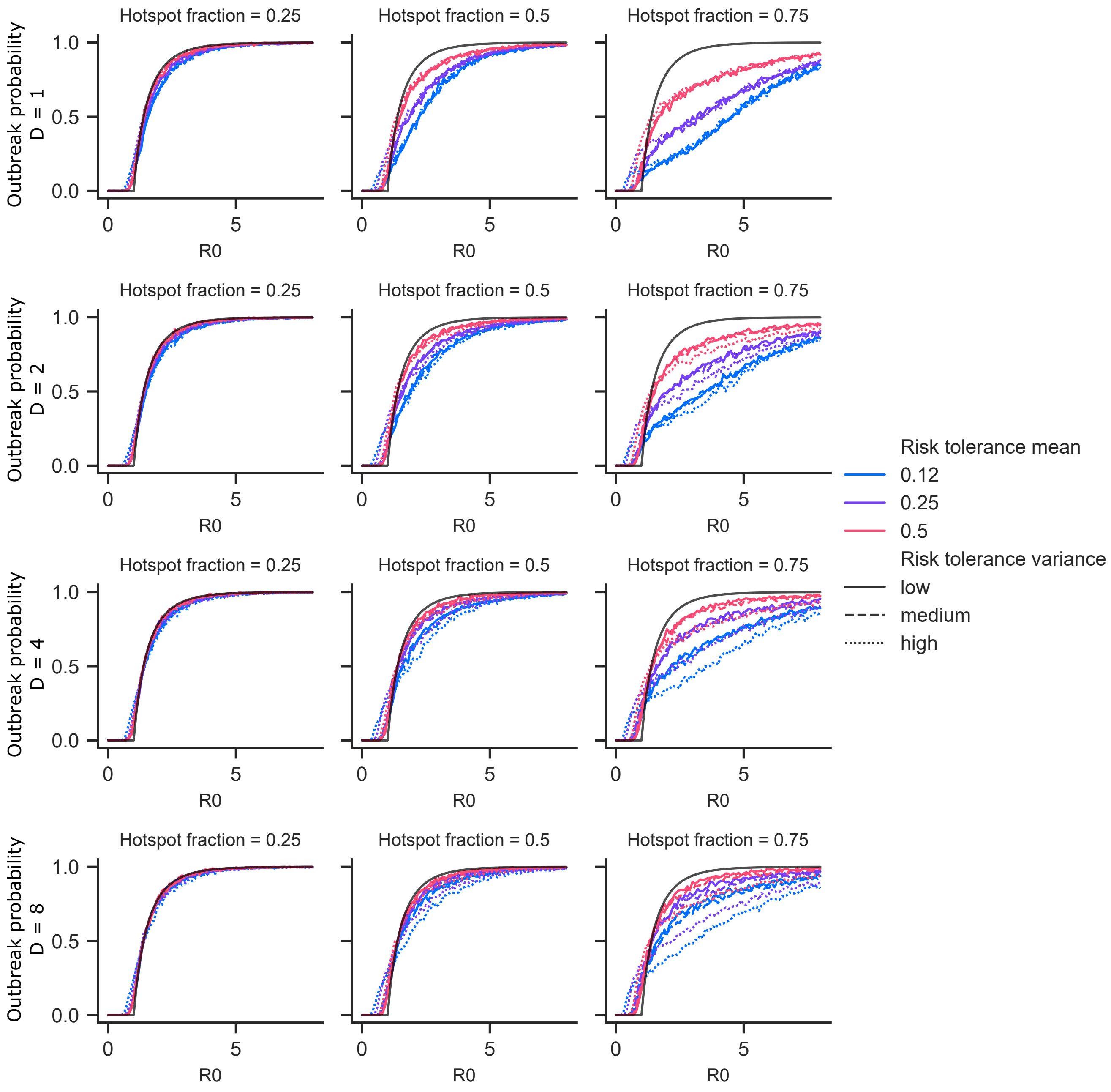

Fig. S3. Outbreak probabilities - extended. On outbreak has occurred if at least 5% of individuals were infected over the course of a simulation. In a homogeneous model, the probability of this occurring increases sharply as a function of R_0 (black lines). Colored lines show outbreak probability across 1,000 trials in the agent-based model as a function of R_0 for 9 different risk tolerance distributions, different columns show varying contributions of hotspot spread to R_0. Rows show results for increasing disease length times D.

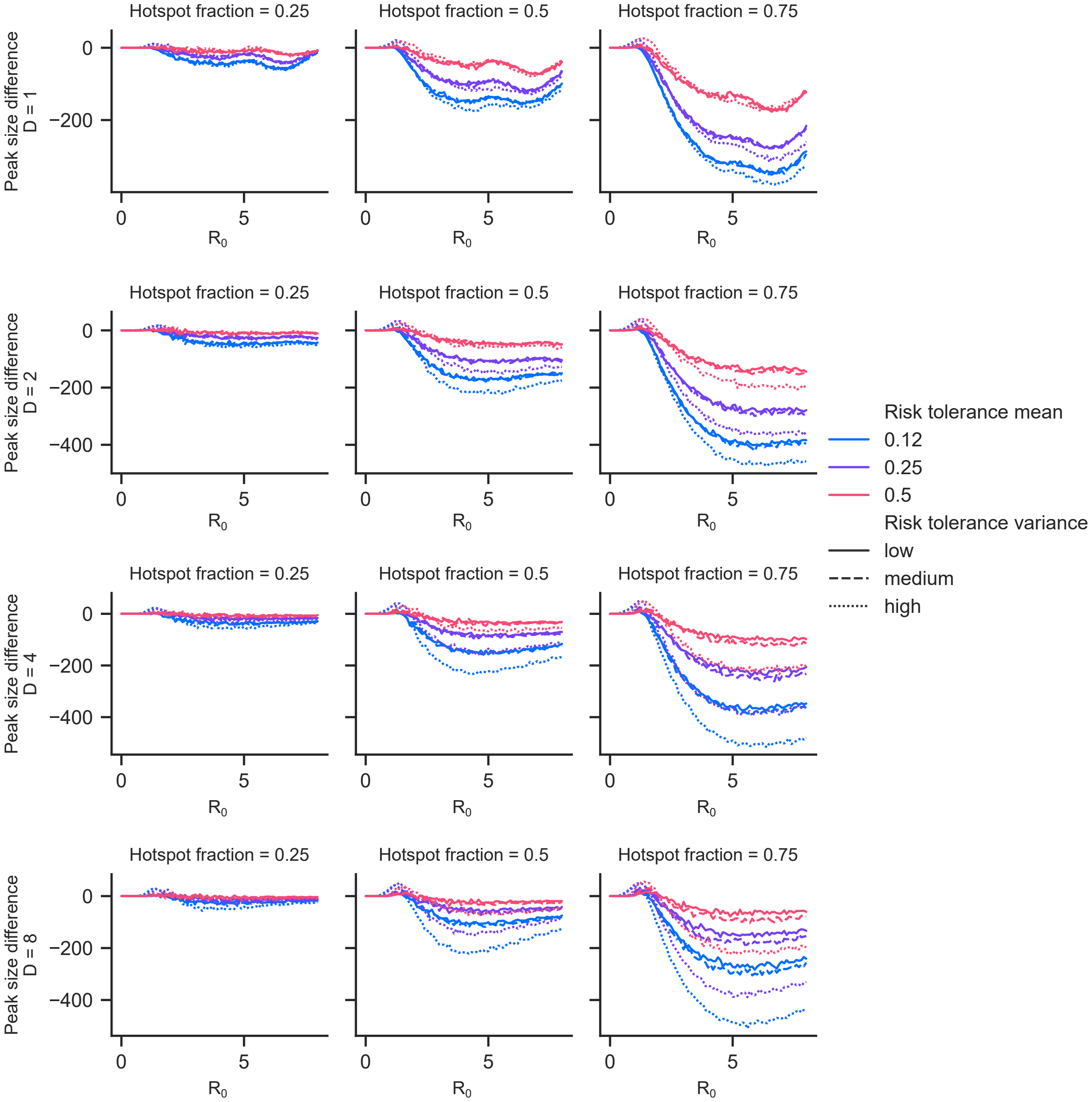

Fig. S4. Peak outbreak sizes - extended. Colored lines show average epidemic size minus the peak predicted by a homogeneous model with the same R_0. "Peak" is the maximum number of infected agents at any given time during a simulation. We limit to simulations in which an outbreak occurred (at least 5% of the population was infected). Rows show results for increasing disease length times D.

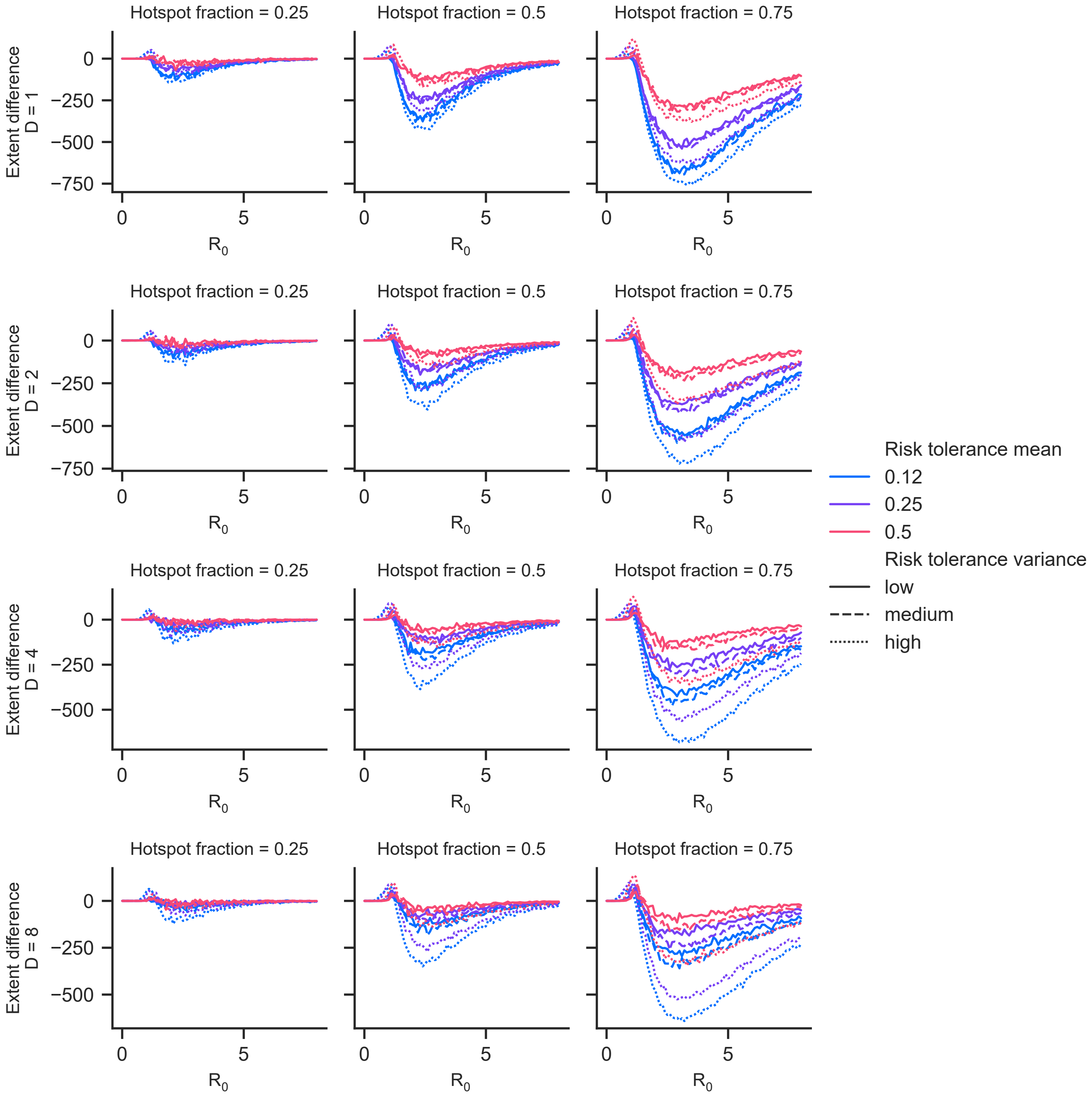

Fig. S5. Outbreak extents - extended. Colored lines show average final size minus the final size predicted by a homogeneous model with the same R_0. "Final size" is the total number of agents experiencing infection during a simulation. We limit to simulations in which an outbreak occurred (at least 5% of the population was infected). Rows show results for increasing disease length times D.

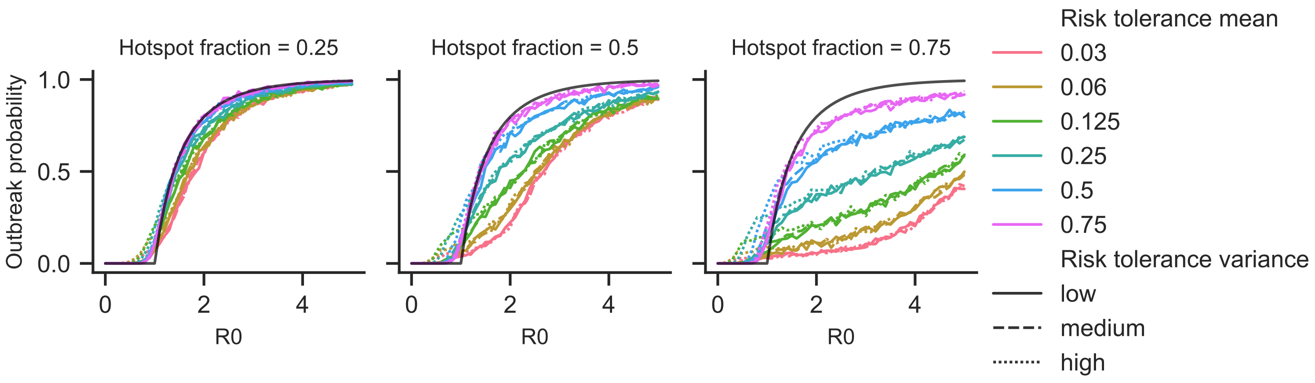

Fig. S6. Outbreak probability - extended. Colored lines show outbreak probability (at least 5% of individuals infected) across 1,000 trials in the agent-based model. Here we consider a wider range of risk tolerance distributions than the same figure in the main text.

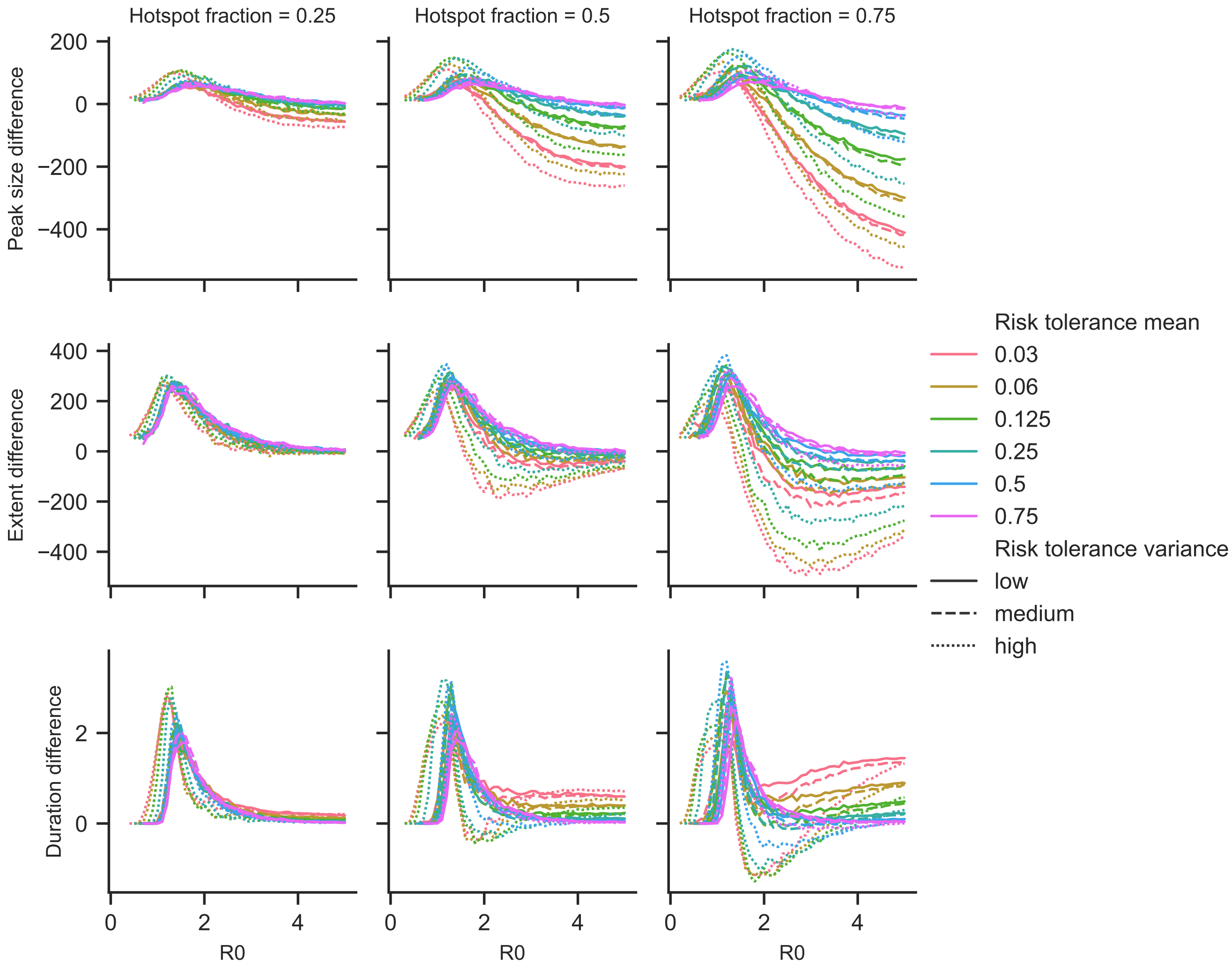

Fig. S7. Outbreak peak, extent and timing - extended. Colored lines show average peak size minus the peak size predicted by a homogeneous model with the same R_0 (Top), average final size minus the final size predicted by a homogeneous model (Middle), and average outbreak duration minus the outbreak duration predicted by the homogeneous model (Bottom). Here we consider a wider range of risk tolerance distributions than the same figure in the main text.
